# The efficacy of pyridostigmine therapy after transurethral resection of prostate in cases with underactive urinary bladder: prospective randomized trial

**DOI:** 10.1007/s00345-025-05842-8

**Published:** 2025-08-02

**Authors:** Khaled Magdy Zeinelabden, Mohamed El-Shazly, Ammar Alorabi, Hossam Kandeel, Baher Salman, Mohammed Aziz

**Affiliations:** 1 Urology Department, Faculty of Medicine, Kafrelsheikh University, Kafrelsheikh, Egypt; 2 Urology Department, Faculty of Medicine, Menoufia University, Shibin El Kom, Egypt

**Keywords:** BPH, TURP, Underactive bladder, Detrusor underactivity, Pyridostigmine

## Abstract

**Background:**

Bladder outlet obstruction mediated underactive bladder represents a challenging condition in which transurethral resection of prostate did not prove to be a sufficient treatment option. Therefore, this study was conducted to evaluate the effects and adverse effects of Pyridostigmine as a treatment for underactive bladder after transurethral resection of prostate.

**Methods:**

This prospective, double-blind, randomized controlled study was conducted between May 2024 and November 2024. Sixty-six patients who had benign prostatic hyperplasia with preoperative underactive bladder and eligible for transurethral resection of prostate were randomized into two groups: the Pyridostigmine group, which received Pyridostigmine 120 mg daily for 3 months postoperatively, and the control group, which received placebo postoperatively. Patients were followed-up for 3 months postoperatively to observe symptom changes, urodynamic changes and adverse effects.

**Results:**

Patient who received Pyridostigmine showed significant improvement compared to the control group patients regarding IPSS score (*p* = 0.001), quality of life (*p* < 0.001), postvoid residual volume (*p* = 0.002), maximum flow rate (*p* < 0.001), contractility index (*p* = 0.001) and postoperative retention incidence (*p* = 0.005). Mild adverse effects were reported in 23.5% of patients who received Pyridostigmine with no reported serious adverse effects.

**Conclusion:**

Pyridostigmine after transurethral resection of prostate in patients with underactive bladder with benign prostatic hyperplasia leads to significant improvements in postoperative subjective and objective outcomes with insignificant adverse effects and wide safety profile making Pyridostigmine a therapeutic option for enhancing bladder function recoverability after transurethral resection of prostate.

## Introduction

Underactive bladder (UAB) is a urological condition characterized by a reduction in the strength and duration of detrusor contraction and consequently, incomplete bladder emptying with/out prolongation while the main pathology of UAB is not well understood [1, 2]. One of the main causes of UAB is prolonged bladder outlet obstruction (BOO) due to prostatic enlargement [3, 4]. To make a diagnosis, it is necessary to perform a pressure-flow study, which can distinguish between decreased maximum flow rate (Q-max) related to BOO with/out poor bladder contractility [5].

Parasympathomimetic agents have been suggested as potential therapeutic options for patients with UAB [6, 7]. However, a recent systematic review on the efficacy and safety of these agents for UAB found that the evidence is still limited, without definitive conclusions on their benefits or adverse effects [8].

One of parasympathomimetic agents is Pyridostigmine, which is commonly used to treat myasthenia gravis and has also been studied for the management of UAB [9, 10]. Patients with UAB after transurethral resection of the prostate (TURP) may still experience recurrent episodes of obstructive lower urinary tract symptoms (LUTS), highlighting the need for effective treatment options in these cases [11].

To address this gap, this study aims to evaluate the efficacy of Pyridostigmine therapy in patients with UAB following TURP, using a prospective randomized trial design.

## Patients and methods

This study was designed as a prospective, double-blind, randomized controlled trial to evaluate the efficacy of Pyridostigmine therapy in patients with UAB following TURP.

The study included male patients aged 45 to 75 years who were admitted to Menoufia university hospitals from May 2024 till November 2024 with benign prostatic hyperplasia (BPH) who were eligible for TURP and had decreased bladder contractility confirmed by urodynamic study. We excluded patients with contraindications to Pyridostigmine, diabetes, or a history of neurological diseases or pelvic surgery.

All patients underwent standard bipolar TURP performed by a single experienced urologist using bipolar system (Olympus, Japan) then the catheter removed at the 3rd day postoperatively. Patients with procedure related complications such as perforation and clot retention were excluded.

Patients were then randomized into two groups; Control group, patient who received placebo oral tablets, twice daily for 3 months postoperatively and Pyridostigmine group, patients who received Pyridostigmine 60 mg oral tablet, twice daily for 3 months postoperatively.

Randomization was performed using a computer-generated random number sequence, and the allocation was concealed using sequentially numbered, opaque, sealed envelopes. Both participants and researchers were blinded to the group allocation.

Participants were followed up at 1st, 2nd and 3rd month intervals after surgery. The following outcomes were measured at each time point: Severity of LUTS by International Prostate Symptom Score (IPSS) and quality of life (QoL), Post-void residual volume (PVR) by ultrasonography while objective outcomes were measured at 3rd month including Q-max by flowmetry and both bladder contractility (bladder contractility index) and detrusor pressure by urodynamic study.

Detrusor pressure (PdetQmax) is defined as the pressure produced by the detrusor muscle at the moment of maximum urine flow during a pressure-flow study while bladder contractility index (BCI) is defined as a numeric value reflecting detrusor contractile strength during voiding, calculated as: BCI = PdetQmax + 5× Qmax. Where PdetQmax is the detrusor pressure at maximum flow, and Qmax is the maximum flow rate. A BCI value helps distinguish between normal, weak, and strong bladder contractility. Decreased bladder contractility was diagnosed based on urodynamic findings following the International Continence Society definitions. Specifically, we used BCI. A BCI < 100 was considered indicative of decreased bladder contractility, in line with established criteria in the literature [1, 12].

The study protocol was approved by the local university ethics committee (Menoufia University Institutional Review Board) under the number 5-2024UROL3 and the study was performed in accordance with the ethical standards as laid down in the 1964 Declaration of Helsinki and its later amendments. All participants were counseled and fully informed about the safety profile of Pyridostigmine, signed a written informed consent and were given schedule for follow up before enrollment. Confidentiality and data protection measures were implemented throughout the study.

All data was collected, tabulated, and statistically analyzed using IBM SPSS software package version 25.0. Categorical data were represented as numbers and percentages. Qualitative data was described as numbers and percentages. The Chi-square test was used for categorical variables. Student’s *t* test, paired sample t-test and Mann–Whitney test were used for quantitative variables. The level of significance was set to 5%.

## Results

Seventy-six BPH patients who were meeting our patient selection criteria were enrolled. Ten patients were excluded throughout the study, while 66 patients were analyzed as demonstrated in CONSORT flow chart.


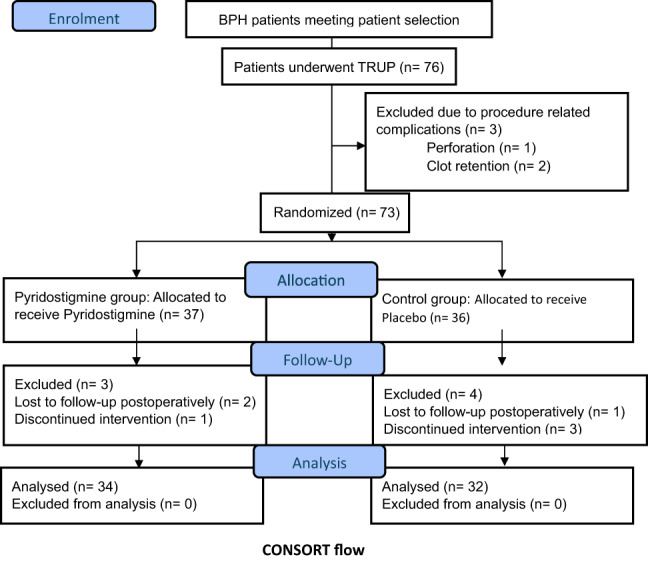


There were non-significant differences between the studied groups regarding demographic and preoperative baseline data (Table 1).

**Table 1:** Comparison between the two studied groups according to demographic and baseline data

	Pyridostigmine group (*n* = 34)	Control group (*n* = 32)
Age, years (mean ± SD)	66.12 ± 4.29	66.06 ± 5.51
BMI, kg/m^2^ (mean ± SD)	26.60 ± 3.62	26.77 ± 3.08
PSA, ng/ml (mean ± SD)	1.92 ± 0.79	1.55 ± 0.86
Prostate size, g (mean ± SD)	83.15 ± 20.42	82.88 ± 20.64
Smoking		
Smoker, n (%)	16 (47.1)	12 (37.5)
Non-smoker, n (%)	18 (52.9)	20 (62.5)
IPSS score, n (mean ± SD)	25.47 ± 4.87	27.91 ± 5.30
IPSS QoL, n (mean ± SD)	3.47 ± 1.19	3.94 ± 0.76
PVR, mL^3^ (mean ± SD)	275.59 ± 57.48	303.13 ± 62.60
Q-max, mL/s (mean ± SD)	6.14 ± 2.25	5.88 ± 2.03
Pressure detrusor, cmH_2_O (mean ± SD)	40.88 ± 13.77	41.25 ± 14.01
Bladder contractility index, n (mean ± SD)	71.62 ± 9.903	70.63 ± 10.39

*CI* confidence interval, *BMI* body mass index, *PSA* prostate specific antigen, *IPSS* International Prostate Symptom Score, *QoL* quality of life, *PVR* post-void residual volume, *Q-max* maximum flow rate

Regarding subjective outcomes (IPSS score, QoL and PVR), there were significant differences between both groups after 1 month, 2 months and 3 months postoperatively except for PVR which showed significant difference only after 2 months and 3 months (Table 2).

Regarding objective outcomes, there were statistically significant differences between both groups regarding Q-max and BCI after three months while there was no significant difference between both groups regarding detrusor pressure. Postoperative retention incidence was significantly higher among the control group (Table 2).

In Pyridostigmine group, there were significant differences between preoperative and 1-month, 2-month and 3-month postoperative outcomes regarding IPSS score, QoL and PVR except for IPSS QoL which did not show significant difference between baseline and after 1 month. Regarding Q-max, detrusor pressure and BCI, there were significant differences between preoperative and after 3 months postoperative (Table 2).

In control group, there were significant differences between preoperative and 1-month, 2-month and 3-month postoperative outcomes regarding IPSS score and PVR while IPSS QoL did not show significant differences. Regarding Q-max, detrusor pressure and BCI, there were significant differences between preoperative and after 3 months postoperative (Table 2).

**Table 2:** Comparison between the studied groups regarding outcomes preoperatively and 1, 2, 3 months postoperatively

	Pyridostigmine group (*n* = 34)	Control group (*n* = 32)	*P* value	Difference (95% CI)
Subjective outcomes preoperatively, 1 month, 2 months and 3 months				
IPSS score, n (Mean ± SD)				
Preoperative	25.47 ± 4.87	27.91 ± 5.30		
After 1 month	14.94 ± 9.73	20.75 ± 12.05	0.035*	− 5.81 (− 11.18, − 0.43)**
After 2 months	9.76 ± 7.87	19.59 ± 12.49	0.001*	− 9.83 (− 14.93, − 4.73)**
After 3 months	9.68 ± 8.12	18.53 ± 13.10	0.001*	− 8.85 (− 14.18, − 3.52)**
	P1 < 0.001*, P2 < 0.001*, P3 < 0.001*	P1 < 0.001*, P2 < 0.001*, P3 < 0.001*		
IPSS QoL, n (mean ± SD)				
Preoperative	3.47 ± 1.19	3.94 ± 0.76		
After 1 month	3.50 ± 1.33	3.72 ± 2.03	0.610	− 0.22 (− 1.06, 0.62)**
After 2 months	1.82 ± 1.53	3.56 ± 2.03	< 0.001*	− 1.73 (− 2.61, − 0.86)**
After 3 months	1.68 ± 1.55	3.47 ± 2.03	< 0.001*	− 1.79 (− 2.67, − 0.90)**
	P1 = 0.845, P2 < 0.001*, P3 < 0.001*	P1 = 0.540, P2 = 0.296, P3 = 0.195		
PVR, mL^3^ (Mean ± SD)				
Preoperative	275.59 ± 57.48	303.13 ± 62.60		
After 1 month	126.56 ± 108.04	168.31 ± 130.14	0.160	− 41.75 (− 100.44, 16.92)
After 2 months	75.15 ± 89.13	162.38 ± 135.16	0.003*	− 87.23 (− 143.21, − 31.25)**
After 3 months	72.09 ± 90.58	162.53 ± 135.01	0.002*	− 90.44 (− 146.67, − 34.21)**
	P1 < 0.001*, P2 < 0.001*, P3 < 0.001*	P1 < 0.001*, P2 < 0.001*, P3 < 0.001*		
Objective outcomes preoperatively, 1 month, 2 months and 3 months				
Q-max, mL/s (Mean ± SD)				
Preoperative	6.14 ± 2.25	5.88 ± 2.03		
After 3 months	11.20 ± 2.16	8.53 ± 2.46	< 0.001*	2.67 (1.53, 3.81)**
P value	< 0.001*	< 0.001*		
Detrusor pressure, cmH_2_O (mean ± SD)				
Preoperative	40.88 ± 13.77	41.25 ± 14.01		
After 3 months	50.26 ± 12.89	47.47 ± 13.77	0.397	2.79 (− 3.75, 9.35)
P value	< 0.001*	0.001*		
Bladder contractility index, n (mean ± SD)				
Preoperative	71.62 ± 9.903	70.63 ± 10.39		
After 3 months	106.29 ± 16.32	90.13 ± 20.58	0.001*	16.16 (7.06, 25.27)**
P value	< 0.001*	< 0.001*		
Retention, n (%)	3 (8.8)	12 (37.5)	0.005*	− 28.7% (− 48.0%, − 9.4%)**

*CI* confidence interval, *SD* standard deviation, *IQR* interquartile range, *P1* p value for comparing preoperative and 1 month postoperative outcome, *P2*, p value for comparing preoperative and 2 month postoperative outcome, *P3* p value for comparing preoperative and 3 month postoperative outcome, *IPSS* International Prostate Symptom Score, *QoL* quality of life, *PVR* post-void residual volume, *Q-max* maximum flow rate

*p < 0.05 is statistically significant; **CI does not contain 0 means difference is statistically significant

Adverse effects were reported in 8 (23.5%) Pyridostigmine group patients including 3 (8.8%) had bloating, 1 (2.9%) had fatigue, 1 (2.9%) had muscle cramps, 1 (2.9%) had diarrhea, 1 (2.9%) had increased salivation and 1 (2.9%) had stomachache. Among control group patients, one patient experienced fatigue.

## Discussion

BPH is considered the most common cause of BOO in elderly men [3, 13]. About 25–30% of patients with BPH also have detrusor underactivity DU, so that deterioration of detrusor muscle contractility is the cause of surgical failure after TURP [12]. The relation of UAB and BOO was documented in many studies [14]. Detrusor contractility declines in long-term BOO and whether relieving BOO surgically is beneficial to this type of patient has been a matter of debate [15].

Drake et al. (2020) concluded that the routine use of urodynamic study in evaluation of uncomplicated LUTS has a limited role and should be selectively used [16]. However, in a study of urodynamically assessed patients preoperatively, patients with DU alone had significantly worse outcome at 12 months compared to patients with BOO with/out DU [17].

Although UAB has no definite protocol for management, UAB management options include mainly conservative methods and clean intermittent catheterization (CIC), pharmacotherapy, neuromodulation, surgical option, and stem cell and gene therapies [4, 18]. CIC is a recommended option for patients who have significant PVR or retention [19].

Based on the fact that longstanding BOO eventually leads to DU and UAB, studies presented surgeries relieving BOO, such as TURP, laser prostatectomy and transurethral incision of the bladder neck, as treatment options [20]. However, the success of surgery in this category of patients is controversial. Kim et al. (2018) emphasized that BPH patients with preoperative UAB has low and limited improvement in IPSS score and Q-max and recommended to exclude these patients from surgical eligibility due to its unbeneficial outcomes [11].

Oral medications used in the UAB vary from alpha-blockers, which reduce urethral outlet pressure to muscarinic agonists, cholinesterase inhibitors, and prostaglandin E2 which enhance bladder contraction. However, none of these agents is completely effective [10]. cholinesterase inhibitors were proved to significantly improve UAB symptoms and decease PVR [21]. Pyridostigmine represents one of the most important pharmacological treatment options for UAB [8].

Pyridostigmine inhibits the breakdown of acetylcholine at the neuromuscular junction promoting signaling. Consequently, it increases the tonus and force of spontaneous contractions of bladder wall [22].

To the best of our knowledge, no prior studies have specifically evaluated the effect of Pyridostigmine as a treatment for UAB post-TURP. Patients with BPH with preoperative UAB were given Pyridostigmine then followed up for 3 months in comparison to a placebo group. There were significant improvements between preoperative and 3 month postoperative outcomes in both groups except for IPSS QoL which showed no significant improvement in the control group.

Tanaka et al. (2006) similarly showed that TURP is an effective surgical procedure for treatment of LUTS/BPH regardless the presence of DU preoperatively [23]. Moreover, Han et al. (2008) found that IPSS score, QoL and PVR significantly improved following TURP in UAB patients with no significant improvement regrading Q-max [24].

Although the previously mentioned studies recommended TURP alone as a treatment option for patients with preoperative DU [23, 24], our study revealed that patient who received Pyridostigmine showed more significant improvement, which tended to increase over time, compared to the control group patients regarding IPSS score, QoL, PVR, Q-max, BCI and postoperative retention incidence.

We likewise managed to examine the correlation between the change in postoperative outcomes and baseline data in both groups. There was a positive significant correlation between IPSS score and age, and between detrusor pressure and BMI while there was a negative significant correlation between IPSS QoL and smoking among patients who received Pyridostigmine. On the other hand, there was positive significant correlations between IPSS score, IPSS QoL and Q-max in arm and PSA in the other arm. There was a negative significant correlation between detrusor pressure and prostate size among patients who received placebo.

To our knowledge, this is the first study to evaluate the effect of Pyridostigmine as a treatment for UAB post-TURP. However, the use of Pyridostigmine as a treatment for UAB was reported in many studies [6, 10]. Since the cost benefit of acetylcholinesterase inhibitors use, specifically Pyridostigmine, has been insufficiently examined and whether its efficacy in treating BOO-mediated UAB justify the burden adverse effects is still questionable, Many urologists tend either to avoid undergoing TURP for patients who have UAB preoperatively or to undergo TURP with just observing and following up the patient postoperatively [25].

A study over 100 myasthenia gravis patients who received Pyridostigmine reported that more than one third of patients experienced one or more side effects. About 30% of these side effects were gastrointestinal, 6% were increased salivation, 3% were urgency and 1% were rash and 1% were blurred vision while stomach pain lead to discontinuation of the drug in one patient [26]. Cholinergic crisis represented as one of the most serious reported side effects that results from Pyridostigmine overdose [27].

Although there is no standardized Pyridostigmine dose for myasthenia treatment, the used dose is usually 180–240 mg daily and may be raised up to 480 mg daily for some cases. This dose is believed to be associated with frequent side effects [28]. These side effects proved to be dose dependent since patients experienced significant lower side effects when dose was reduced [9, 28]. The incidence of adverse effects, especially cholinergic ones, was not correlated to the duration of use [27].

Adverse effects reported in 23.5% of our study patients were all mild, controlled by symptomatic treatment and did not lead to discontinuation of treatment except for one patient who experienced severe diarrhea. One of our control patients claimed to have fatigue which can be attributed to nocebo effect [29]. We believe that the low incidence and mildness of adverse effects is because of the low dose used. The used dose of 120 mg daily proved to cause significant improvement with safety optimization.

Although our study is a novel study that allowed the evaluation of Pyridostigmine effects and side effects in treatment of UAB after TURP, there were a few limitations that needed to be acknowledged. One of these limitations is the relatively small sample. Moreover, short term follow-up of our study was one of our limitations especially the long-term safety of Pyridostigmine which ought to be reported.

We recommend more studies to evaluate the effect of Pyridostigmine over the long-term in BOO mediated UAB after TURP.

We believe that our study may encourage urologists to do TURP for BOO patients with UAB after proper counselling.

## Conclusion

Pyridostigmine after TURP in patients with UAB with BPH leads to significant improvements in postoperative subjective and objective outcomes with insignificant adverse effects and wide safety profile making Pyridostigmine a therapeutic option for enhancing bladder function recoverability after TURP.

## Data Availability

No datasets were generated or analysed during the current study.

## Abbreviations

BCI: Bladder contractility index
BMI: Body mass index
BOO: Bladder outlet obstruction
BPH: Benign prostatic hyperplasia
CIC: Clean intermittent catheterization
DU: Detrusor underactivity
IPSS: International Prostate Symptom Score
LUTS: Lower urinary tract symptoms
PSA: Prostate specific antigen
PVR: Post-void residual volume
Q-max: Maximum flow rate
QoL: Quality of life
TURP: Transurethral resection of the prostate
UAB: Underactive bladder
